# Retraction Note: TRIM58 functions as a tumor suppressor in colorectal cancer by promoting RECQL4 ubiquitination to inhibit the AKT signaling pathway

**DOI:** 10.1186/s12957-026-04545-7

**Published:** 2026-08-26

**Authors:** Naizhi Sun, Jiacheng Shen, Yuhua Shi, Biao Liu, Shengguo Gao, Yichuan Chen, Jinwei Sun

**Affiliations:** Department of General Surgery, North Hospital of Yancheng Third People’s Hospital, The Yancheng School of Clinical Medicine of Nanjing Medical, Theater Road No. 75, Tinghu District, Yancheng, Jiangsu Province 224000 China

**Retraction Note: World Journal of Surgical Oncology 21, 231 (2023)**

**https://doi.org/10.1186/s12957-023-03124-4**

The Editor-in-Chief has retracted this article.

After publication, concern was raised regarding the image overlap with a previously published article [1]. The authors were contacted to clarify the issue but they did not respond.

Concern:

Figure 6 A in this article appears to overlap with Fig. 5B of [1].

On behalf of all authors, Jinwei Sun stated that they agree with this retraction.
